# Biomimetic and NOS‐Responsive Nanomotor Deeply Delivery a Combination of MSC‐EV and Mitochondrial ROS Scavenger and Promote Heart Repair and Regeneration

**DOI:** 10.1002/advs.202301440

**Published:** 2023-06-06

**Authors:** Ning Zhang, Mengkang Fan, Yongchao Zhao, Xiaolong Hu, Qiongjun Zhu, Xiaolu Jiao, Qingbo Lv, Duanbin Li, Zheyong Huang, Guosheng Fu, Junbo Ge, Hongjun Li, Wenbin Zhang

**Affiliations:** ^1^ Department of Cardiology Key Laboratory of Cardiovascular Intervention and Regenerative Medicine of Zhejiang Province Sir Run Run Shaw Hospital Zhejiang University School of Medicine Hangzhou 310016 China; ^2^ Department of Cardiology Affiliated Hospital of Nantong University Nantong 226001 China; ^3^ Department of Cardiology Zhongshan Hospital Fudan University Shanghai Institute of Cardiovascular Diseases Shanghai 200032 China; ^4^ College of Pharmaceutical Sciences Zhejiang Laboratory of Systems and Precision Medicine Zhejiang University Hangzhou 310012 China

**Keywords:** biomimetic, cardiac repair, drug delivery, extracellular vesicle, nanomotors

## Abstract

Mesenchymal stem cell‐derived extracellular vesicle (MSC‐EV) is shown to promote cardiac repair, however, it still falls short in initiating myocardia proliferation restart. In this regard, ROS‐induced DNA damage and responses are the culprit of cellcycle arrest. Here, this work constructs a hybrid cell‐derived extracellular vesicle that is composed of MSC and macrophage membranes and encompasses MitoN, a ROS scavenger, to boost the healing of the heart. The MitoN, a NAD(P)H mimic, could target the mitochondrial to eliminate the ROS resuming the arrested cell cycle. The hybrid extracellular vesicle (N@MEV) could respond to the inflammatory signals generated during myocardial injury and thus enable superior targeting and enrichment to the location of the damage. L‐arginine, which could be catalyzed by NOS and ROS into NO and SO provide a driving force, is immobilized within the vesicle (NA@MEV) to further enhance the N@MEV's potential to penetrate the cardiac stroma. In combination with multiple mechanisms, NA@MEV increased heart function 1.3‐fold EF% versus MSC‐EV in mouse myocardial injury model. A more in‐depth mechanistic study found that the NA@MEV could modulate M2 macrophage; promote angiogenesis; reduce DNA damage and response, and thereby restart cardiomyocyte proliferation. Thus, this combined therapy shows synthetic effects in heart repair and regeneration.

## Graphical abstract

MSC‐EV enveloping NADH mimic are engineered into macrophage biomimetic and NO‐driving nanomotor. The nanomotor colonizes at injured vascular and then catalyze arginine into NO to derived toward deep tissues. Through the combination, NADH mimic alleviates ROS‐induced DNA damage and DDR, and restarts myocardium proliferation; MSC‐EV increase such proliferation, and elicits immunoregulation and proangiogenesis, which synergistically promote heart repair and regeneration.

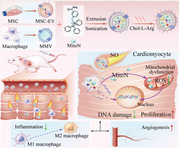

## 1 Introduction

Myocardial infarction (MI) and ischemic‐reperfusion injury (I/R) lead to a high risk of developing heart failure. For the reestablishment of lost function caused by permanent cell death, mesenchymal stem cell‐derived extracellular vesicle (MSC‐EV) has been extensively examined^[^ ^1^ ^]^ and determined its effectiveness in cardiomyocyte protection, immune modulation and angiogenesis.^[^ ^2^, ^3^, ^4^ ^]^ However, the efficacy of heart regeneration has come under scrutiny due to conflicting and inconsistent results across multiple laboratories. Moreover, low homing efficiency poses a significant challenge as the treatment fails to target and repair the affected heart regions effectively. These impediments have impeded the translation of this therapy into clinical practice.

Mammalian cardiomyocytes lose their proliferative ability at birth and during ischemic episodes. This is due to oxidative damage, which is associated with cell cycle arrest, triggered by reactive oxygen species (ROS) produced by mitochondrial oxidative metabolism. Cardiomyocytes experience a burst of DNA damage and DNA damage response (DDR) activation, eventually leading to cell‐cycle arrest.^[^ ^5^, ^6^, ^7^ ^]^ Reducing oxidative damage derived from mitochondria prolongs neonatal cardiomyocyte regeneration and restarts adult cardiomyocyte proliferation. At the same time, mitochondrial dysfunction is caused by ROS‐induced lipid peroxidation, mitochondrial DNA damage, and uncoupling of oxidative phosphorylation, which contributes to ROS formation through feedback mechanisms.^[^ ^8^ ^]^ In light of the high energy consumption in the adult heart, we hypothesized that stem cell‐EV synergizing mitochondrial‐targeted ROS scavenging should be a viable strategy for promoting cardiomyocyte proliferation and tissue repair without interfering with energy production.

N‐substituted aromatic ring is a critical part of the structure of endogenous antioxidants, NAD(P)H/NAD(P)+, that undergoes oxidative reaction before becoming NAD(P)+.^[^ ^9^ ^]^ 1,4‐dihydropyridines are a group of Ca2+ antagonists that share this structure and possess potent ROS scavenging ability, whereas they cannot dissolve and accumulate into mitochondria largely without a targeting compound.^[^ ^10^ ^]^ Herein, a NAD(P)H mimic (MitoN) was synthesized where dihydronicotinamide was conjugated with triphenylphosphonium (TPP) to drive toward the mitochondrial matrix and scavenge ROS.^[^ ^11^, ^12^ ^]^ The MitoN reduced ROS accumulation in mitochondrion and associated DNA damage and acted as the key to the cardiomyocyte cell cycle. When cooperated with the MSC‐EV, the proliferation efficiency was significantly improved. Besides that, a macrophage‐derived cell membrane was fused with MSC‐EV for biomimetic targeting delivery of the combinations to the ischemic‐injured tissue by responding to the inflammatory signal.^[^ ^13^, ^14^ ^]^ To achieve deeper penetration of macrophage biomimetic MSC‐EV into the injured heart tissue, cholesterol‐conjugated arginine was incorporated into the biomimetic MSC‐EV, which created a biomimetic and nitric oxide synthase (NOS)‐responsive nanomotor that can react to high‐level and spatially distributed NOS and ROS. By catalyzing arginine into NO, this nanomotor offers the necessary driving force to penetrate deeper tissues within the injured hearts (Scheme).^[^ ^15^, ^16^ ^]^

In this study, we investigated the effects of our fabricated NA@MEV in heart repair and regeneration. The combined therapy had synergistic effects, including immune regulation, proangiogenesis, and mitochondrial protection. Notably, the combination of MitoN and MSC‐EV was able to alleviate mitochondrial ROS‐induced DNA damage and DDR, leading to enhanced myocardial cell cycle reentry and proliferation compared to MSC‐EV monotherapy. We used a macrophage biomimetic and NOS‐responsive nanomotor delivery system to deliver MitoN‐cooperated MSC‐EV to the ischemic‐injured hearts and found that the nanomotor NA@MEV accumulated the most in the deep zones of the heart tissue (2.6‐fold of MSC‐EV, 1.3‐fold of MEV), significantly promoting myocardial cell cycle reentry and proliferation and improved heart functions.

## 2 Results

### 2.1 Preparation and Characterization of NA@MEV

**Scheme 1:**
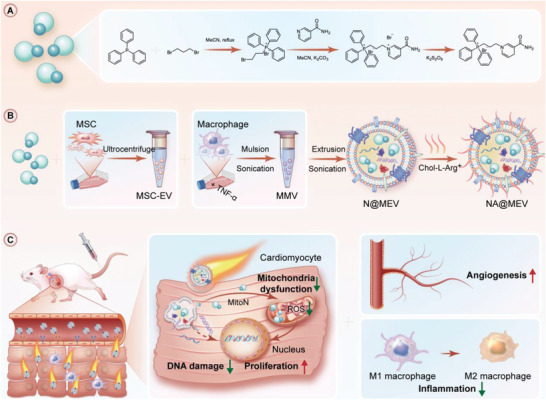
Preparation and therapeutic effects of NA@MEV. A) Chemical synthesis of mitochondrial‐targeted ROS‐scavenging NAD(P)H mimic MitoN. B) Fabrication of macrophage biomimetic and NO‐driving nanomotor NA@MEV. C) Therapeutic effects of NA@MEV for I/R injured hearts, including mitochondrial protection, decreased DNA damage and myocardial proliferation reentry, immunomodulation, and proangiogenesis.

**Figure 1:**
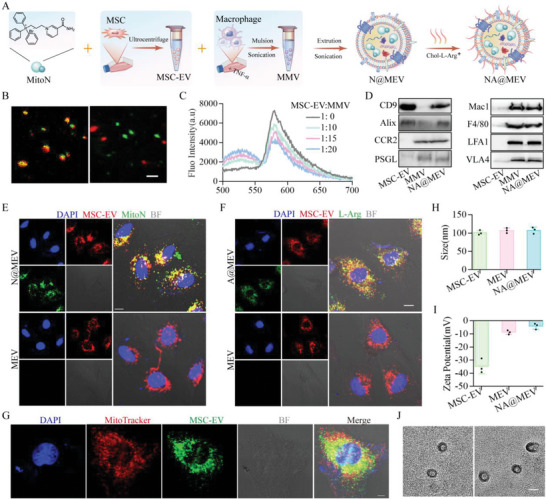
Preparation and characteristics of NA@MEV. A) Scheme of the fabrication process of NA@MEV. B) Confocal fluorescence images of NA@MEV (left) and the direct mixture of MSC‐EV and MMV (right). bar = 1 µm. C) The FRET analysis determined their fluorescence spectra after membrane fusion procedures. D) Western blot confirmed typical proteins of MSC‐EV, MMV, NA@MEV. E) Confocal fluorescence co‐localization of N@MEV (upper) and MEV(lower) confirmed the loading of MitoN within N@MEV. bar = 20 µm. F) Confocal fluorescence co‐localization of A@MEV (upper) and MEV(lower) confirmed the L‐Arg on the L@MEV. bar = 20 µm. G) Confocal fluorescence co‐localization of mitochondrion and N@MEV confirmed the MitoN targeting to mitochondrion. bar = 10 µm. H,I) Size (H) and *ζ* potential (I) of MSC‐EV, MMV and NA@MEV. *n* = 3. J) TEM of MEV(left) and NA@MEV(right). bar = 100 nm.

The mitochondrial ROS scavenger MitoN was synthesized as the **Scheme** **1** HNMR and 13 CNMR spectrum shown in Figures S1 and S2, Supporting Information, demonstrated that the MitoN was obtained. Macrophage biomimetic and NO‐driven MSC‐EV nanomotor NA@MEV was then fabricated by a 3‐steps procedure (**Figure** **1**A). First, as our lab reported, MSC‐EV was collected from the culture medium by an ultracentrifuge method.^[^ ^17^, ^18^ ^]^ Meanwhile, macrophage membrane vesicles (MMV) were isolated from TNF‐*α* pre‐activated RAW 264.7 cells. Then macrophage biomimetic MSC‐EV, namely MEV, was fabricated after membrane fusion.^[^ ^17^ ^]^ When the MitoN was pre‐added, drug‐loaded macrophage biomimetic MSC‐EV was obtained and named N@MEV. To verify the fusion between MSC‐EV and MMV, fluorescent colocalization under confocal confirmed the fluorescence signal overlay of two examples after fabricating procedures rather than direct mixture (Figure 1B). Next, a FRET assay was conducted and further revealed the fusion between MSC‐EV and MMV, where fluorescence at 583 nm gradually decreased during MMV: MSC‐EV increased, and shared high FRET efficiency under the ratio of 10:1 (Figure 1C). Meanwhile, the use of 0.2 µm polycarbonate membrane during extrusion did not cause additional damage to the integrity after determining the remaining total RNA and DNA contents, that MEV preserved 93% RNA and 94% DNA compared to MSC‐EV (Figure S3, Supporting Information). Furthermore, protein expression was analyzed by WB, and it found that typical proteins of EVs and macrophages were well inherited to MEV (Figure 1D). Finally, N@MEV was modified with (Cholesterol‐)L‐arginine on the surface following sonication and stirring, and thereafter the macrophage biomimetic and NO‐driving MSC‐EV nanomotor (NA@MEV), was obtained. To confirm whether MitoN was loaded into MEV, MitoN, and DiD‐labeled MSC‐EV were used to fabricate N@MEV, and then cocultured with H9C2. The cell uptake under confocal was observed and showed colocalization of two fluorescence signals indicating successful loading of MitoN within N@MEV (Figure 1E). Similarly, (Cholesterol‐NBD) L‐arginine was used to modify MEV and cell internalization was also observed. Results suggested the successful insertion of arginine on the surface of A@MEV under our methods when compared with MEV (Figure 1F). As a proof of concept, Mito‐Tracker (Red) was used to indicate mitochondria, and after internalized by H9C2, MitoN in NA@MEV actually was accumulated to mitochondria as shown by a significant overlay of signals between MitoN and Mito‐Tracker in Figure 1G. In the end, the size and Zeta‐potential of NA@MEV were determined, and results showed that the fusion with MMV did not lead to a significant change to the size and morphology of MSC‐EV, according to DLS and TEM results (98.06 nm vs 102.59 nm, MSC‐EV vs NA@MEV, Figure 1H–J). Whereas the *ζ* potential of NA@MEV decreased significantly compared to that of MSC‐EV (−34.83 mV vs −3.8 mV, MSC‐EV vs NA@MEV, Figure 1H), which indicates better stability in the blood and successful loading of MitoN.

### 2.2 N@Mev Decreases Ros Burst and Mitochondrial Dysfunction in Cardiomyocyte

**Figure 2:**
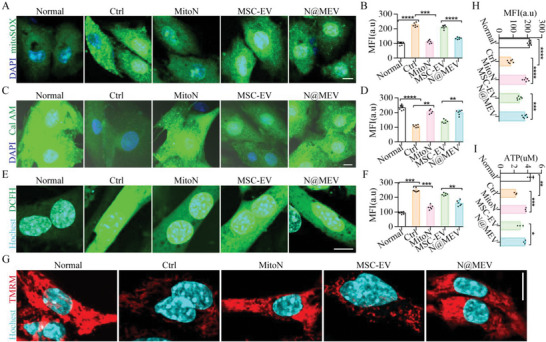
ROS scavenging and mitochondrial protection of N@MEV. A,B) H9C2 mitochondrial ROS burst after oxidative stress and respective treatments was indicated by mitoSOX (A) and fluorescently quantified (B). Green, mitoSOX. bar = 10 µm, *n* = 6. C,D) H9C2 mitochondrial permeability transition pore was indicated (C) and quantified (D) by Calcein AM. Green, Calcein AM. bar = 10 µm, *n* = 6. E,F) ROS accumulation in adult mouse cardiomyocytes after oxidative stress and respective treatments was indicated (E) and quantified (F) by DCFH‐DA. Green, ROS. bar = 10 µm, *n* = 6. G,H) Adult cardiomyocytes mitochondrial membrane potential was indicated (G) and quantified (H) by TMRM. Red, TMRM. bar = 10 µm, *n* = 6. I) ATP volume in adult cardiomyocytes after oxidative stress and respective treatments was determined by a kit (*n* = 3). *p < 0.05, **p < 0.01, ***p < 0.001, ****p < 0.0001.

We first detected ROS burst after oxidative stress to examine whether N@MEV exerts a combined therapy in mitochondrial antioxidant and cardiac repair. As shown in **Figure** **2**A,B, ROS accumulation in H9C2 mitochondria was indicated by mitoSOX and showed that N@MEV and MitoN significantly decreased mitochondrial ROS burst. In contrast, MSC‐EV showed poor effects, which further demonstrated the mitochondrial‐targeted ROS scavenging ability of MitoN within N@MEV. Then mitochondrial injury was compared by probe Cal AM (Figure 2C,D), N@MEV and MitoN treatments alleviated H9C2 mPTP opening after H_2_O_2_ exposure. To further demonstrate myocardial protection of N@MEV, adult myocardium was cocultured under H_2_O_2_ and respective treatments. DCFH‐DA staining showed N@MEV and MitoN could also significantly decrease cardiomyocyte total ROS accumulation, as compared to MSC‐EV treatment (Figure 2E,F). Meanwhile, mitochondrial dysfunction reflected by MMP was investigated by TMRM probe and further proved that N@MEV, as well as MitoN could protect cardiomyocyte mitochondrial function after oxidative stress (Figure 2G,H). As a result, ATP synthesis ability of myocardium after H_2_O_2_ exposure and treatments was determined in Figure 2I, and ATP level results were consistent with the above conclusions that N@MEV and MitoN protected cardiomyocyte from mitochondrial injury and metabolic disorders. In addition, and preserved energy synthesis ability upon H_2_O_2_ exposure.

### 2.3 N@Mev Alleviates Cardiomyocyte DNA Damage and Promotes Proliferation

**Figure 3:**
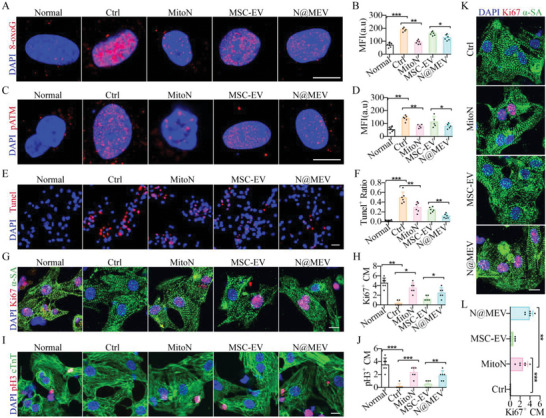
Neonatal cardiomyocyte DNA damage and proliferation. A,B) Neonatal cardiomyocyte (P1 CM) DNA damage after oxidative stress and treatments was determined at 8‐oxoG (A) and further quantified (B). Red, 8‐oxoG. bar = 10 µm, *n* = 6. C,D) P1 CM DDR was determined at pATM expression by IF staining (C) and further quantified (D). Red, pATM. bar = 10 µm, *n* = 6. E,F) P1 CM apoptosis was determined by Tunel staining (E) and quantification (F). Red, Tunel. bar = 10 µm, *n* = 6. G,H) P1 CM proliferation was investigated by Ki67 and *α*‐SA IF staining (G) and further quantified (H). Green, *α*‐SA; Red, Ki67. bar = 10 µm, *n* = 6. I,J) P1 CM proliferation was also investigated by pH3 and cTnT IF staining (I) and quantified (J). Green, cTnT; Red, pH3. bar = 10 µm, *n* = 6. K,L) P7 CM proliferation after respective treatments investigated by Ki67 and *α*‐SA IF staining (K) and further quantified (L). Green, *α*‐SA; Red, Ki67. bar = 10 µm, *n* = 6. *p < 0.05, **p < 0.01, ***p < 0.001.

Mitochondrial ROS‐induced DNA damage can be detrimental to the cell cycle and proliferation of cardiomyocytes (CMs). Therefore, we examined whether MSC‐EV combined with ROS scavenging could promote cardiomyocyte proliferation. When treated with N@MEV and MitoN, cardiomyocyte DNA damage as indicated by 8‐oxogG was significantly decreased, while MSC‐EV showed a poorer effect (**Figure** **3**A,B). Upon DNA damage, DDR was further evaluated at pATM expression, a DNA damage sensor and repair pathway. Results revealed a much lower level of DDR in cardiomyocyte after being treated with N@MEV or MitoN, while MSC‐EV‐treated CM showed high‐level pATM (Figure 3C,D). These data demonstrated the stronger antioxidant activity of N@MEV rather than MSC‐EV, in mitochondrial ROS accumulation and nuclear DNA oxidative damage. As a result, H_2_O_2_‐induced CMs apoptosis was measured by Tunel and showed that N@MEV exerted a synergistic effect in cardiomyocyte protection compared to MitoN and MSC‐EV (Figure 3E,F). The findings indicated that N@MEV exhibited a dual effect of cell protection inherited from MSC‐EV and anti‐oxidative properties of MitoN, thereby providing a combined therapeutic approach for cell protection.

After that, cell proliferation after oxidative stress was investigated. H_2_O_2_ significantly decreased the proportion of proliferative P1 CMs, while MitoN, MSC‐EV, and N@MEV could preserve cell cycle progress and proliferation when Ki67‐ and pH3‐positive CMs were observed and numbered under confocal (Figure 3G–J). However, when P7 CMs were treated the same without H_2_O_2_, only N@MEV‐ and MitoN‐endowed CMs showed ability to reenter the cell cycle and proliferate as Ki67 and pH3 indicated. During the study, it was observed that N@MEV treatment of P7 cardiomyocytes resulted in a synergistic effect. When MSC‐EV was combined with MitoN, there was more significant increase in the proportion of proliferative cardiomyocytes compared to either treatment alone. Interestingly, it was found that using MSC‐EV and MitoN separately also had a similar effect in increasing the proportion of proliferative cardiomyocytes (Figure 3K,L, Figure S4A,B, Supporting Information). Cell mitosis in the treatment groups was further evaluated by Auro B staining in Figure S4C,D, Supporting Information, which showed that a spot of CMs underwent mitosis after N@MEV and MitoN treatments. Western blot determination at the expression of Ki67 and pH3 of these CMs further confirmed above results (Figure S4E,F, Supporting Information).

In addition, when exposed to H_2_O_2_, N@MEV‐, and MitoN‐treated P7 CMs also obtained the ability to proliferate and showed synergistic effect in N@MEV group (Figure S4G,H, Supporting Information), which altogether demonstrated that H_2_O_2_‐induced oxidative injury leads to CM cell cycle arrest, whereas MitoN, MSC‐EV, and N@MEV can to some extent preserve neonatal CM regeneration. Of note, the N@MEV treatment was found to be more effective than monotherapy, as it could prolong the 7 day‐window of mammalian CM regeneration, promote arrested cell cycle restart and proliferate; the MitoN is crucial to restart cell cycle through alleviating ROS‐induced DNA damage/DDR pathway activation. On the other hand, MSC‐EV monotherapy was unable to restart cell cycle, but it was observed to improve the regeneration of cardiac muscle when used in combination with MitoN,. In the end, the tubule‐forming of endothelium and immunomodulation of macrophage were measured in vitro. Results showed that MSC‐EV, as well as N@MEV, increased tubule density (Figure S4I,J, Supporting Information); decreased M1 but increased M2‐associated genes expression in macrophages after TNF‐*α* stimulation (Figure S4K, Supporting Information), which suggested N@MEV still possess the potential of pro‐angiogenesis and immunomodulation after engineered from MSC‐EV.

### 2.4 Na@Mev Nanomotor Target to Deep Myocardial Tissue after I/R Injury

**Figure 4:**
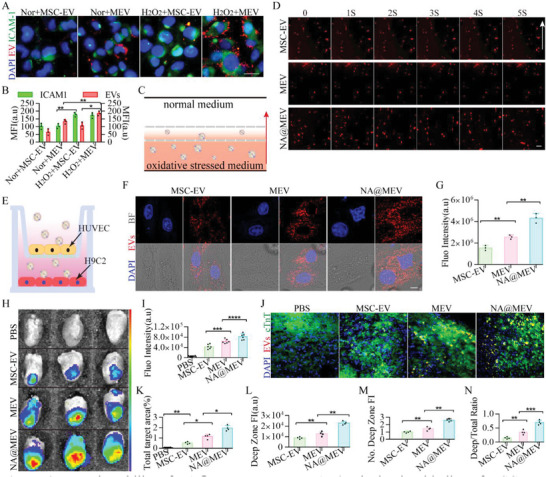
Targeting ability of NA@MEV nanomotor. A,B) The in vitro binding of MSC‐EV, MEV to oxidative stressed HUVEC and H9C2 were imaged (A) after immunofluorescent stained with ICAM‐1 and further quantified (B). Green, ICAM‐1; Red, MSC‐EV or MEV. bar = 20 µm, *n* = 3. C) Scheme illustrating the construction of motion tracking system. Upper chamber, normal condition medium; middle chamber, motion‐observation; lower chamber, HUVEC oxidative stress‐conditioned medium added with MSC‐EV, MEV, and NA@MEV. Red arrow: motion direction of respective nanoformations. D) Motion tracking images of MSC‐EV, MEV, and NA@MEV under confocal was acquired at indicated times. White arrow: motion direction of respective nanoformations. bar = 1 µm. E) Scheme illustrating the construction of Transwell. F,G) Images and quantification of H9C2‐internalized respective formulations. H,I) IVIS images (H) and quantification (I) of respective formulations accumulated in the hearts after i.v. injected after I/R. J,K) Heart section images (J) and quantification (K) of respective formulations totally accumulated in the heart tissues. Green, cTnT; Red, respective formulations. bar = 20 µm, *n* = 4. L,M) Fluorescent quantitation (L) and normalization (M) of respective formulation showing NA@MEV accumulated the most in heart deep‐injured zones (low‐cTnT areas) than MSC‐EV and MEV. *n* = 4. N) Fluorescent quantitation ratio of deep zone/total showed NA@MEV mainly accumulated in injured deep zones. *n* = 4. *p < 0.05, **p < 0.01, ***p < 0.001, ****p < 0.0001.

To examine whether macrophage biomimetic MEV can target injury‐activated endothelium, we first conducted a cell binding experiment and found that upon H_2_O_2_ exposure, adhesive molecules ICAM‐1 upregulated, meanwhile MEV rather than MSC‐EV significantly bond to endothelium and showed colocalization with ICAM‐1(**Figure** **4**A,B), which is coincident to previous work.^[^ ^17^ ^]^ Next, we investigated whether NA@MEV can work as a nanomotor under oxidative stress condition. After culturing with HUVEC and H9C2 oxidative stress conditioned medium, only NA@MEV lead to a remarkable increase in the NO concentration (Figure S5, Supporting Information), which indicated that HUVEC and H9C2 could produce NOS and ROS as fuel for NA@MEV nanomotor. Then, under our motion tracking system, we observed that NA@MEV could actively move with a tropism to the lower NOS and ROS side; whereas, MSC‐EV and MEV did not show ability of active motion (Figure 4C,D). Finally, a Transwell was established where HUVEC in the upper‐ and H9C2 in the lower chamber was pretreated with H_2_O_2_ and then was refreshed with NA@MEV, MEV or MSC‐EV in the upper chamber (Figure 4E). Under confocal, H9C2 uptake of respective examples was observed and showed that NA@MEV was internalized the most and the second is MEV rather than MSC‐EV (Figure 4F). Meanwhile, cells were recorded by fluorescence microplate (Figure 4G), and further demonstrated the conclusion that A@MEV could bind to injury‐activated endothelium and then work as a nanomotor moving toward subendothelial injured cardiomyocytes.

Proofing of concept, these examples were intravenously injected to mice 24 h after I/R. Through IVIS and fluorescence quantification (Figure 4H,I), we found that NA@MEV nanomotor significantly accumulated in the injured heart. Compared to MSC‐EV, the macrophage biomimetic and NO‐forced NA@MEV nanomotor could prominently and accurately target to I/R injured hearts (threefold). Tissue sections after being stained with cTnT were further analyzed and revealed the distribution of these formulations that, NA@MEV nanomotor not only accumulated the most in heart tissues than other formulations (Figure 4J,K), it also remarkably distributed to deep injured zones (low‐cTnT areas), where its fluorescent intensity is 2.59‐fold and 1.79‐fold of MSC‐EV and MEV respectively (Figure 4L,M). Moreover, the deep targeting ratio (deep zone/total accumulation) was analyzed and further demonstrated that NA@MEV nanomotor accumulated mainly (70%) in deep zones, which is 4.6‐fold of what MSC‐EV did (Figure 4N). These results demonstrated that NA@MEV nanomotor can target to myocardial deep‐injured tissues through two forces including macrophage biomimetic adhesion^[^ ^19^, ^20^ ^]^ and NO‐driving.^[^ ^16^, ^21^ ^]^ Finally, the distribution in other organs was also examined (Figure S6, Supporting Information), and similar to the MSC‐EV, NA@MEV also distributed to the liver and lung.

### 2.5 Na@Mev Nanomotor Decreases Myocardial Injury and Restarts Proliferation after I/R

**Figure 5:**
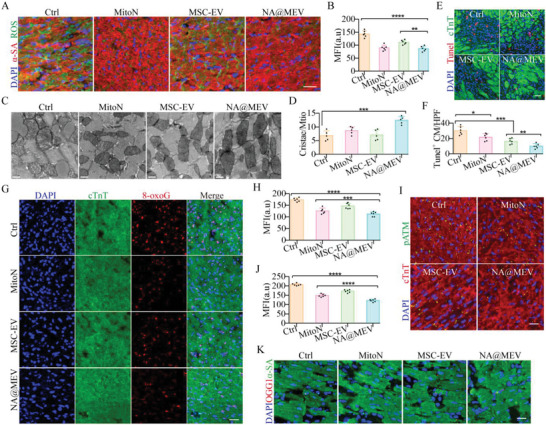
Myocardial oxidative damage after I/R and treatments. A,B) Cardiomyocytes ROS burst indicated by Rhb123 (A) and quantification (B) 7 days after I/R and treatments. Green, ROS; Red, *α*‐SA. bar = 20 µm, *n* = 6. C,D) Cardiomyocyte mitochondrial damage was observed under TEM (C) and further evaluated at mitochondrial cristae density (D). bar = 0.5 µm, *n* = 5. E,F) Cardiomyocyte apoptosis was determined by Tunel staining (E) and further quantified (F). Green, cTnT; Red, Tunel. bar = 20 µm, *n* = 6. G,H) Cardiomyocyte DNA damage was investigated by 8‐oxoG IF staining(G) and further quantified (H). Green, cTnT; Red, 8‐oxoG. bar = 20 µm, *n* = 6. I–K) Cardiomyocyte DDR was investigated at pATM (I, Green, pATM; Red, cTnT) or OGG1(K, Green, *α*‐SA; Red, OGG1) expression by IF staining, and further quantified (J). bar = 20 µm, *n* = 6. *p < 0.05, **p < 0.01, ***p < 0.001, ****p < 0.0001.

Upon I/R injury, ROS volume in myocardium was first evaluated by RhB123 probe 7 days‐after I/R. As indicated by *α*‐SA, myocardial ROS level in MitoN‐ and NA@MEV‐treated groups were much lower than that of MSC‐EV and control groups (**Figure** **5**A,B), which can be attributed mainly to MitoN within the NA@MEV. Consequently, mitochondrial morphology was observed under TEM, and NA@MEV‐treated myocardial mitochondrion suffered less swelling and preserved more cristae compared to other groups (Figure 5C,D). Cell apoptosis indicated by Tunel and cTnT staining also confirmed the cell protection effect of NA@MEV, MSC‐EV and MitoN (Figure 5E,F). On the other hand, 8‐oxoG assessment after I/R and treatments showed potent effects of NA@MEV in decreasing DNA oxidative damage (Figure 5G,H). pATM expression measurement also showed that the DDR was consistently decreased when treated with NA@MEV nanomotor (Figure 5I,J), whereas another pathway involving DNA repair, OGG1, did not show differences (Figure 5K).

**Figure 6:**
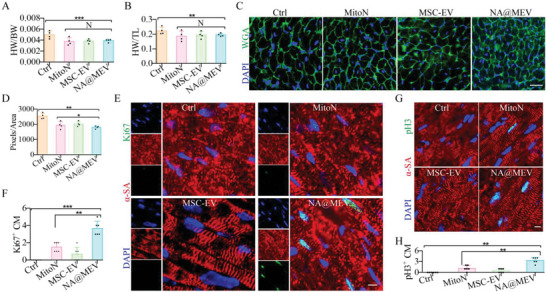
Myocardial regeneration after I/R and treatments. A,B) Mice heart weight to body weight ratio(A) or heart weight to tibia length ratio (B) was determined respectively 4 weeks after I/R and treatments (*n* = 6). C,D) Myocardial cell size was indicated by WGA staining (C) and further quantified (D). Green, WGA. bar = 50 µm, *n* = 4. E,F) Myocardium proliferation was determined at Ki67+ CM by IF staining (E) and further quantified (F). Green, Ki67; Red, *α*‐SA. bar = 20 µm, *n* = 6. G,H) Myocardium proliferation indicated by pH3+ CM (G) and quantification (H). Green, pH3; Red, *α*‐SA. bar = 20 µm, *n* = 6. *p < 0.05, **p < 0.01, ***p < 0.001.

28 days after treatments, heart weight to body weight ratio and heart weight to tibia length ratio were measured and showed that all three treatments of MitoN, MSC‐EV and NA@MEV nanomotor alleviated myocardium hypertrophy (**Figure** **6**A,B). WGA staining and quantification further determined cell size differences between groups, NA@MEV nanomotor significantly decreased cell size and thus indicated more cell volume than other groups (Figure 6C,D). As a result, myocardial regeneration was investigated, and we found that NA@MEV nanomotor treatment increased Ki67‐ and pH3‐posistive myocardium proliferation than MitoN (Figure 6E–H). In contrast, MSC‐EV treatment showed weak effects in promoting proliferation reentry. These results altogether demonstrated that MitoN‐exerted antioxidant activity work as the key to restart cell cycle and when used in combination with MSC‐EV, the cell proliferation can be significantly increased. Furthermore, when the combined therapy was delivered to heart deep tissue after I/R, as NA@MEV nanomotor performed, the efficiency of heart regeneration has been increased comparing to routine antioxidant supply.

### 2.6 Na@Mev Nanomotor Promotes Heart Injury Repair and Functional Recovery

**Figure 7:**
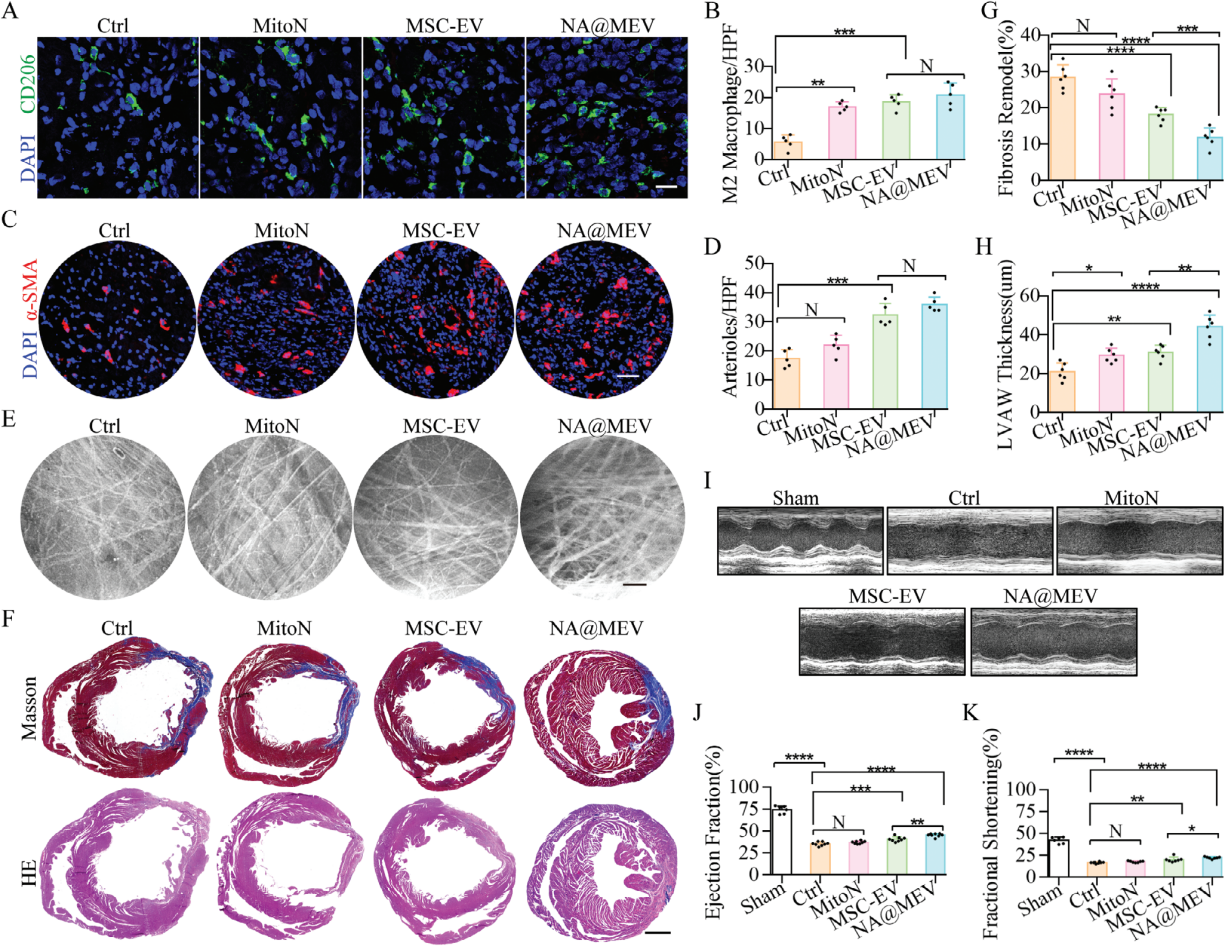
Heart repair and function after I/R and treatments. A,B) IF staining (A) and quantification (B) of mice heart M2 macrophages identified by CD206 7 days after treatments. Green, CD206. bar = 10 µm, *n* = 5. C,D) *α*‐SMA IF staining (C) identified mice heart arterioles 4 weeks after I/R and treatments, and the microvessel density was quantified (D). Red, *α*‐SMA. bar = 50 µm, *n* = 5. E) Heart microcirculation perfusion was observed after i.v. administrated fluorescein sodium under IVCS. bar = 1 mm. F–H) The fibrosis and necrosis were observed after Masson and HE staining (F) and further quantified showing alleviated fibrosis remodeling (G) and preserved LVAW thickness (H) after treatments. bar = 50 µm, *n* = 6. I–K) Cardiac function was determined by echocardiography (I) and quantified (J, EF; K, FS) after treatments. *n* = 7 or 8. *p < 0.05, **p < 0.01, ***p < 0.001, ****p < 0.0001.

To assess the inflammation response during heart repair after I/R, heart sections from mice were collected 7 days after receiving respective treatments. The findings revealed that NA@MEV and MSC‐EV considerably boosted the quantity of anti‐inflammatory macrophages (M2), as evidenced in (**Figure** **7**A,B). This outcome highlights the immunomodulatory attributes of NA@MEV derived from MSC‐EV. Meanwhile, MitoN treatment increased M2 macrophages, which might be attributed to decreased ROS. It was reported that ROS formation is exacerbated in inflammatory macrophages (M1) and act as an important mediator of inflammation pathways, hampering M1‐M2 polarization transformation.^[^ ^22^, ^23^ ^]^ On the other hand, heart microvessel density and microcirculation were investigated through *α*‐SMA immunostaining and perfusion imaging under IVCS. As shown in Figure 7C,D, MSC‐EV and NA@MEV markedly increased arterioles density and MitoN treatment did not show a significant effect. Furthermore, microcirculation perfusion indicated by fluorescein sodium under IVCS further confirmed the proangiogenesis potential of MSC‐EV and NA@MEV, and consequently improved microcirculation perfusion (Figure 7E), which in total demonstrated the combining therapy exerted by NA@MEV in heart injury repair including immunomodulation and angiogenesis. As a consequence, the fibrosis remodeling of heart tissue through Masson and HE staining exhibited that NA@MEV significantly decreased fibrosis volume and preserved LVAW thickness comparing to control and MSC‐EV groups (Figure 7F–H). Finally, as shown in Figure 7I–K, heart function was assessed by echocardiography and showed that the NA@MEV nanomotor‐exerted combining therapy preserved the most of heart function compared to MSC‐EV and MitoN. This was reflected by EF% (35.5 vs 37.2 vs 40.8 vs 45.5, Ctrl vs MitoN vs MSC‐EV vs NA@MEV) and FS% (16.7 vs 17.6 vs 20.1 vs 22.3, Ctrl vs MitoN vs MSC‐EV vs NA@MEV). Altogether, these results demonstrated that macrophage biomimetic and NO‐driven nanomotor deep targeting strategy can efficiently deliver mitochondrial ROS scavenger‐loaded MSC‐EV to ischemic injured heart tissue; and more importantly, the combined therapy exerted by NA@MEV prove to be efficient in short‐ and long‐term heart repair including immunomodulation, angiogenesis and functional recovery. When it comes to myocardial regeneration, mitochondrial‐ROS scavenging could restart cell cycle while MSC‐EV could improve such regeneration progression when synergistically applied.

### 2.7 Biosafety Assessment

The cell toxicity of MitoN has been determined in previous study and demonstrated satisfactory safety in cell viability even at 100 µm.^[^ ^9^ ^]^ Then, the immune response after treatments was determined by quantifying serum specific IgM and IgG, which represent acute and chronic antibody respectively, and FCM results did not suggest potential immune response against MSC‐EV and NA@MEV treatments (Figure S7A, Supporting Information). On the other hand, we investigated histology changes by HE and caspase‐3 IHC staining, but, we did not observe any obvious toxic changes among treatment groups (Figure S7B,C, Supporting Information). Clinically, heart attacks generally occur following micro‐thrombus formation in the coronary, that herein we evaluated thrombogenic risk of these treatments by a platelet aggregation test.^[^ ^24^ ^]^ The results showed that platelets aggregated maximally 85% after preincubated with NA@MEV and stimulated by thrombin, without comparable significances versus other treatments (Figure S7D,E, Supporting Information), indicating slight influences to platelet functions of these treatments.

## 3 Discussion and Conclusion

Heart attacks generally lead to permanent myocardium loss and cardiac dysfunction. Investigators have determined MSC‐EV been effective in angiogenesis and anti‐inflammation. However, it has only moderate potency in supporting cardiomyocytes proliferation, which can significantly hinder progress toward repairing the heart. However, recent evidence suggests that cell cycle arrest can be attributed to oxidant injury from mitochondrial resources. In coronary obstruction and reperfusion, mitochondrial oxidative metabolism dysfunction and resultant ROS accumulation leads to extensive damages, particularly to DNA, ultimately resulting in the arrest of cardiomyocyte cell cycle. Targeting mitochondrial ROS provides an approach to prevent DNA damage and facilitate cell proliferation through the provision of genetic materials.

Herein, we conducted a combined therapy to improve therapeutic efficacy in heart regeneration and repair, where MSC‐EV and mitochondrial‐targeted antioxidants were used as a combination and further delivered to heart injured tissue through macrophage biomimetic and NO‐driving strategy. Our results showed that the microenvironment of injured tissues can be utilized to design cascade‐targeting platforms for deep regions and subcellular structure targeting. The biomimetic targeting has been successful in colonizing at injury‐activated coronary and subendothelial matrix, but is feeble to reach deep tissues. The extensive and upregulated NOS in injured tissues, however, provided us opportunity to drive MSC‐EV toward injured myocardial tissue in this study. Through our fabricated NA@MEV, it elicited synergic effects in heart repair and regeneration, not only showed MSC‐EV‐associated mechanisms of immunoregulation and proangiogenesis, but also alleviated mitochondrial ROS‐induced DNA damage and DDR. An important finding is that when used in combination, MitoN worked as the key to myocardial cell cycle reentry and once restarted, the MSC‐EV significantly increased such proliferation after ischemic injury which further emphasized the importance of DNA status for cell regeneration. By reducing DNA damage and DDR in the upstream of cell cycle arrest, myocardium proliferative capacity was extended in mouse under our conditions which is consistent to others.^[^ ^5^ ^]^ This suggested that intervention in oxidative metabolism and associated ROS formation is feasible to promote heart regeneration at least partially through signals involving in DNA damage.

Regarding the synthetic results of the combined therapy in myocardial proliferation, however, the mechanisms behind these results should be further addressed. For example, what contents of MSC‐EV involved and the interaction between MSC‐EV and MitoN behind the augmented cell proliferation need more efforts to target. Stem cell‐EVs have been widely demonstrated proregenerative, but MSC‐EV monotherapy turned out to be weak. This further identified that the DNA are the critical substances for myocardial proliferation and should be suitable or ready to enter cell cycle and proliferate.

To load drugs into EV, several approaches have been achieved but still face significant challenges that their widespread use is limited.^[^ ^25^, ^26^ ^]^ Studies to date have offered methods in EV‐clearing or detecting by electrostatic interaction between EV membrane and nanoparticles.^[^ ^12^ ^]^ Inspired by which, in this study, TPP, an extensively used group in mitochondrial‐tracking, was used to drive attached NAD(P)H mimic into EV, and intriguingly, these cargos further targeted toward mitochondria matrix and exhibited antioxidant activity once internalized. This is important to extend therapeutic use of NAD(P)H derivates given their difficulty to enter cell and localize to mitochondrion. Of note, these NAD(P)H mimics showed potent antioxidant activity in mitochondria, and leaded to decreased damage to mitochondrial function and nuclear DNA.

## 4 Experimental Section

### Materials and Chemistry Synthesis

Triphenylphosphine, 1,3‐dibromopropane, nicotinamide, MeCN, K_2_CO_3_, and K_2_S_2_O_8_ were purchased from Shanghai Aladdin Biochemical Technology Co. Cholesterol‐L‐arginine and cholesterol‐NBD‐arginine were purchased from Ruixi Biotech Co. Mitochondrial targeted ROS scavenger MitoN was chemically synthesized by a previous method with modifications and were detailly described in supporting materials.

### Cell Culture and Fabrication of Na@Mev

Rat bone marrow MSC were maintained under the conditions in previous study and passage 3–6 was used to isolate MSC‐derived exosomes (MSC‐EVs) by an ultracentrifuge method.^[^ ^17^, ^27^ ^]^ Macrophage cell line, RAW264.7, was pretreated with TNF‐*α* (50 µg mL^−1^) to stimulate inflammatory targeting property before isolating membrane vesicles (MMV) by a commercial kit and sonication. Macrophage biomimetic MES‐EV (MEV), was fabricated by the previous method,^[^ ^17^ ^]^ that macrophage membrane vesicles and MSC‐EV (10:1, m:m) were mixed and stir‐incubated under 37 °C for 15 min before several extrusions through membrane to allow membrane fusion. Meanwhile, to load MitoN into MEV, excessive MitoN (1 mg mL^−1^) was pre‐added before above procedures and followed by ultrafiltration to remove excessive MitoN. The NO‐driven nanomotor NA@MEV was obtained after modifying N@MEV with (Cholesterol‐)L‐arginine (0.1%, wt%) through 5 min mild sonication and 37 °C 6 h stirring, and ultrafiltration.

### Characteristics of Na@Mev

The membrane fusion between macrophage mimics and MSC‐EV was examined by fluorescent co‐localization. MMV and MSC‐EV were pre‐labeled with DiO (Sigma) and DiD (Sigma), respectively. And then, the fabricated MEV were observed under confocal microscope (Olympus, Japan) to verify fusion. Next, FRET assay was conducted as previous method where fluorescence spectrum was recorded from 500 to 700 nm with exciting at 470 nm.^[^ ^17^, ^18^ ^]^ Typical proteins involving in macrophages inflammatory infiltration was identified on MMV and MEV by Western Blot (WB). At last, NA@MEV was characterized by dynamic laser scattering (DLS, Malvern) and further observed under TEM.

### Drug Loading and Mitochondrial Targeting Verification

To examine whether MitoN was loaded into N@MEV, MSC‐EV was pre‐labeled with DiD to fabricate N@MEV. Then 1 × 10^6^ H9C2 cells were maintained in 6‐well, and the uptake of which by H9C2 cells was observed under confocal microscope after 2 h incubation (10 µg mL^−1^). The surface modification of Arginine onto N@MEV was examined with similar procedures where NBD‐Arginine was used to fabricate NA@MEV and H9C2 cells was also observed after 2 h incubation (10 µg mL^−1^) to allow internalization. The MitoN targeting to mitochondrion after internalization was further investigated using Mito‐Tracker after 4 h incubation with H9C2 cells at 10 µg mL^−1^.

### The Antioxidant and Pro‐Proliferation Activity of Na@Mev In Vitro

To examine the antioxidant activity of NA@MEV, 1 × 10^6^ H9C2 cells or cardiomyocytes that were extracted from P1, P7 primary neonatal (CM) or adult C57BL/6 mice, were treated with 100 µm H_2_O_2_ to induce oxidative stress and cocultured with 10 µg mL^−1^ MSC‐EV, NA@MEV, or 100 µm MitoN respectively, for 12 h. Then ROS burst and scavenging in cells was assessed by mitoSOX (Invitrogen) and DCFH‐DA (beyotime, China) probe and quantified by fluorescent microscope. Mitochondrial injury and dysfunction regarding as mitochondrial permeability transition pore (mPTP), mitochondrial membrane potential (MMP) after treatments were detected by a Calcein AM (Cal AM, beyotime, China) and TMRM (Invitrogen) probe, respectively. Normal or control groups were maintained under standard condition or 100 µm H_2_O_2_ constantly. The function of mitochondria in producing ATP after treatments was assessed by an ATP Assay Kit (beyotime, China). Cell oxidative base modification of DNA, 8‐oxo‐7,8‐dihydroguanine (8‐oxoG, Invitrogen) and phosphorylated ataxia telangiectasia mutation (pATM, Invitrogen), were immunoassayed respectively to evaluate ROS‐induced DNA damage and DDR. The proteins involved in proliferation, G2‐M progression (phosphorylated histone H3 Ser10, pH3, Huabio, China) and late G1‐M progression (Ki67, Invitrogen) were determined by immunofluorescent (IF) staining and WB. As a result, cell apoptosis was investigated by TUNEL staining (Beyotime, China) and quantified under fluorescent microscope.

### Cell Binding and Targeting Experiments

HUVEC cells (1 × 10^6^) were seeded in 6‐well plates with or without H_2_O_2_ (100 µm) for 10 h. Then, cells were washed and incubated with 5 µg mL^−1^ DiD‐labeled MSC‐EV and MEV, at 4 °C for 10 min. Then, cells were washed, the typical adhesive protein ICAM‐1 was identified by IF staining. Meanwhile, HUVEC and H9C2 were pretreated with H_2_O_2_, and their conditioned medium was collected and cultured with MSC‐EV, MEV, and A@MEV for 2 h before NO determination by a kit. Then, a motion tracking system was established where the upper chamber filled normal condition medium; the lower chamber filled oxidative stressed HUVEC conditioned medium, and was added with MSC‐EV or MEV or A@MEV; the middle chamber used to observe active motion under microscope. Finally, HUVEC in the upper were and H9C2 in the lower chamber were maintained in a transwell (1 × 10^5^), until the HUVEC being contact‐inhibiting before treated with MSC‐EV, MEV, A@MEV, following H_2_O_2_ exposure, for 5 h. Then internalization of respective samples by H9C2 cells were observed and quantified. 24 h after I/R operation, mice were intravenously injected with 200 µL DiD‐labeled MSC‐EV, MEV, A@MEV, or PBS. Accumulation of respective examples in mouse hearts was investigated 2 h after administration by the IVIS system and was further assessed after heart sections were stained with anti‐cTnT antibody (Invitrogen). The distribution in other major organs, including the liver, spleen, lung, kidney, and brain was further quantified.

### Experimental Animals and Treatment Assignment

C57B/L mice (20 ± 2 g) were purchased from the Shanghai SLAC Laboratory Animal, Ltd. Animal experiments were approved by the Ethics Committee of Zhongshan Hospital, Fudan University, Shanghai, China (NO.2021007). Mouse myocardial I/R was induced via left anterior descending coronary artery ligation for 60 min and confirmed by ST‐segment characterized electrocardiogram and left ventricle color alteration. Mice were randomly divided into three groups and subjected to various treatments: control group, 100 µL saline was IV injected following I/R on the days 1, 2, and 3; MSC‐EV, NA@MEV group,100 µL (10 µg) respective samples were injected at the indicated times; MitoN group was given 1 mg kg^−1^ MitoN. For the sham group, mice were exposed to sham operation and administration with equal amounts of PBS.

### Cardiomyocyte Oxidative Damage and Proliferation Assessment

The oxidative stress after myocardial I/R was evaluated when heart sections were obtained 5 days after treatments, and Rhd123 and anti‐cTnT antibody were used to indicate ROS in myocardium. Meanwhile, cardiomyocyte mitochondrial morphology and structure was investigated by TEM. Cell injury and apoptosis was evaluated by tissue TUNEL and cTnT immunostaining. Molecules involving in DDR pathway, including 8‐oxog, pATM and OGG1 (CST), were detected by IF staining with specific antibodies, to evaluate cardiomyocyte DNA damage and DDR after treatments. 28 days after treatments, heart weight to body weight ratio, or to tibial length ratio and WGA stain were conducted to compare cardiomyocyte cell size and number. As a result, regenerative potential of cardiomyocytes was further examined by costaining cTnT and pH3 or Ki67 antibody, followed by observation and quantification under microscope.

### Cardiac Repair and Function Assessment

10 h before and 4 weeks after treatments, mice were anesthetized with low‐dose isoflurane, and cardiac function was evaluated by transthoracic echocardiography (Visual Sonics, Vevo 770), where left ventricular ejection fraction (LVEF) and fraction shortening (LVFS) were calculated in six consecutive cardiac cycles, following M‐mode traces at the papillary muscle level. Then, 1 mg kg^−1^ fluorescein sodium salt was IV injected after anesthesia and the coronary microcirculation was observed and compared under an in vivo confocal system (IVCS, MR‐solution, France). Mouse hearts were harvested and cut into 5‐µm sections, fibrosis extension, and infarct size were measured after Masson and HE staining. The arterioles were identified after *α*‐SMA immunostaining to quantify microvessel density (MVD). To investigate immune competence, macrophages in the border zone were identified, among which, M2 was indicated with anti‐CD206 antibody (Abcam).

### Statistical Analysis

The data are presented as the mean ± SD. Statistical analyses were performed with GraphPad Prism (version 7.0). Comparisons between any two groups were analyzed using the two‐tailed unpaired Student's *t‐*test, whereas comparisons between more than two groups were conducted using one‐way analysis of variance procedures. Differences were considered statistically significant when *p* < 0.05.

## Conflict of Interest

The authors declare no conflict of interest.

## Supporting information

Supporting Information

## Data Availability

The data that support the findings of this study are available on request from the corresponding author. The data are not publicly available due to privacy or ethical restrictions.
