# Supplementary material for: Biomimetic and NOS‐Responsive Nanomotor Deeply Delivery a Combination of MSC‐EV and Mitochondrial ROS Scavenger and Promote Heart Repair and Regeneration
Source: Adv Sci (Weinh). 2023 Jun 6;10(21):2301440. doi: 10.1002/advs.202301440 (PMC10375188; doi:10.1002/advs.202301440)
Supplement: Supplementary file 1 — Supporting Information [file ADVS-10-2301440-s001.pdf]

ADVANCED SCIENCE
Open Access

Supporting Information

for *Adv. Sci.*, DOI 10.1002/advs.202301440

Biomimetic and NOS-Responsive Nanomotor Deeply Delivery a Combination of MSC-EV and Mitochondrial ROS Scavenger and Promote Heart Repair and Regeneration

Ning Zhang, Mengkang Fan, Yongchao Zhao, Xiaolong Hu, Qiongjun Zhu, Xiaolu Jiao, Qingbo Lv, Duanbin Li, Zheyong Huang, Guosheng Fu, Junbo Ge, Hongjun Li* and Wenbin Zhang**

Supporting Information

Biomimetic and NOS-responsive nanomotor deeply delivery a combination of MSC-EV and mitochondrial ROS scavenger and promote heart repair and regeneration

Ning Zhang#, Mengkang Fan#, Yongchao Zhao#, Xiaolong Hu, Qiongjun Zhu, Xiaolu Jiao, Qingbo Lv, Duanbin Li, Zheyong Huang, Guosheng Fu, Junbo Ge, Hongjun Li*, Wenbin Zhang**

Methods and Materials

Chemical synthesis: To a 100mL round-bottomed flask equipped with a magnetic stirrer, a mixture of Compound 1 (2.6 g, 10mmol) and compound 2 (2.4 g, 12 mmol) in MeCN (50 mL) was stirred at 80 oC for overnight. The suspension was cooled to room temperature, ether (150 mL) was added. and a white solid was produced and filtered through a sand core funnel to yield crude product 3 (4.2 g, 91%). Compound 3 (4.2g, 9 mmol), and compound 4 (9 mmol), K2CO3 (20 mmol) and MeCN (100 mL) were added into a 250ml flask equipped with a magnetic stirred. The mixture was gently stirred for 12 h under 80 oC. A white solid was produced and filtered through a sand core funnel to yield compound 5 (4.6 g, 88%), which was directly used in the next reaction without further purification. Then the compound 5 (4.6 g) was dissolved in H2O (100 mL), and Na2S2O4 (4.16g, 24 mmol) was added into the system, adjusting pH to 8-9 by Na2CO3 powder. The mixture was placed in dark and stirred for 4h at room temperature. The resulting suspension was filtered and 60ml Methanol was added, the HCl gas was insterted until the solid was totally dissolved in methanol. The solvent was removed to give a yellow solid-compound 1 (3.3 g, yield 72%).

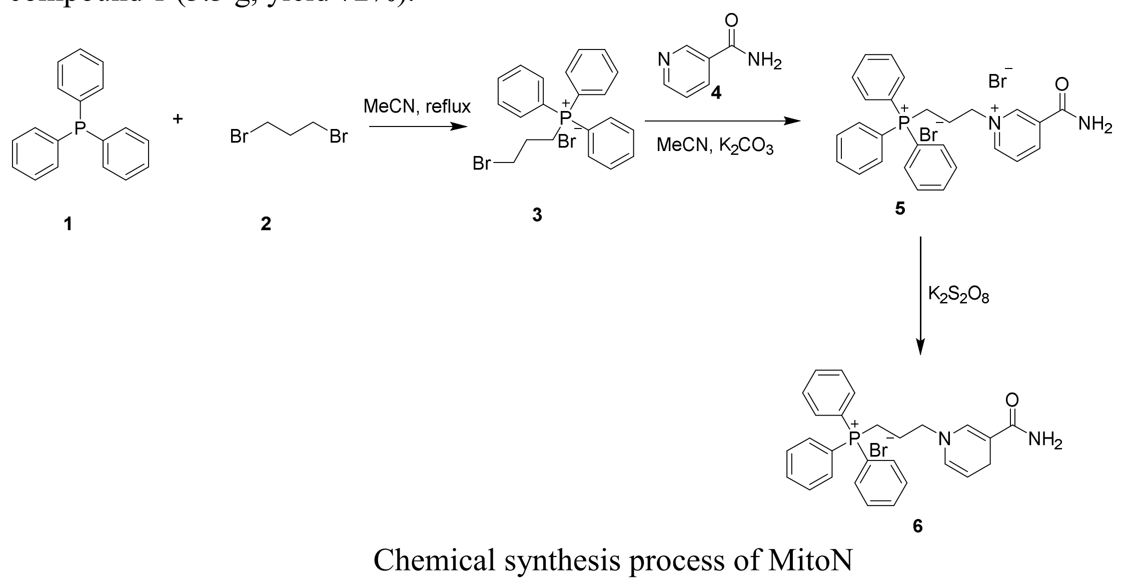

Chemical synthesis process of MitoN

Tube formation experiments: To measure the proangiogenic effects of MSC-EV and N@MEV, human umbilical vein endothelial cells (HUVECs, ATCC, USA) were maintained using endothelial cell growth medium (ECM, ATCC, USA), and treated with MitoN, MSC-EV and N@MEV for 24h. Afterwards, $1.5x10^5$ cells were seeded in 96-well plates that were precoated with 80ul Matrigel and maintained with serum-free medium for 4h. Then, cells were stained with Calcein, and the tubular structures were observed and quantified under microscope.

Biosafety Verification: Regarding immune safety, randomized C57B/L mice were treated with 100 µl (10 µg) MSC-EV and NA@MEV once a week for four times. To examine immune response, mouse serum was collected and incubated with MSC-EV and NA@MEV. Then, all samples were examined by FCM following incubation with rat anti-mouse IgG. Meanwhile, histology changes in major organs were studied by HE and IHC staining with anti-caspases3 antibody. The platelet aggregation test was conducted to investigate thrombotic risk of these treatments. Briefly, mouse platelets were isolated from whole blood to obtain platelet-rich plasma (PRP), then washed and suspended in Tyrode's Buffer (TB), containing Apyrase (1:2000) to inhibit endogenous ADP, at 300×10^9/L to obtain washed platelets (WP). Thereafter, 200 µl WP containing 10 µl MSC-EV or NA@MEV was incubated at 37°C for 10 mins, before triggered by thrombin (0.05U/ml) and platelet aggregation determination by a platelet lumiaggregometer (Chrono-Log, Model 700).

Results

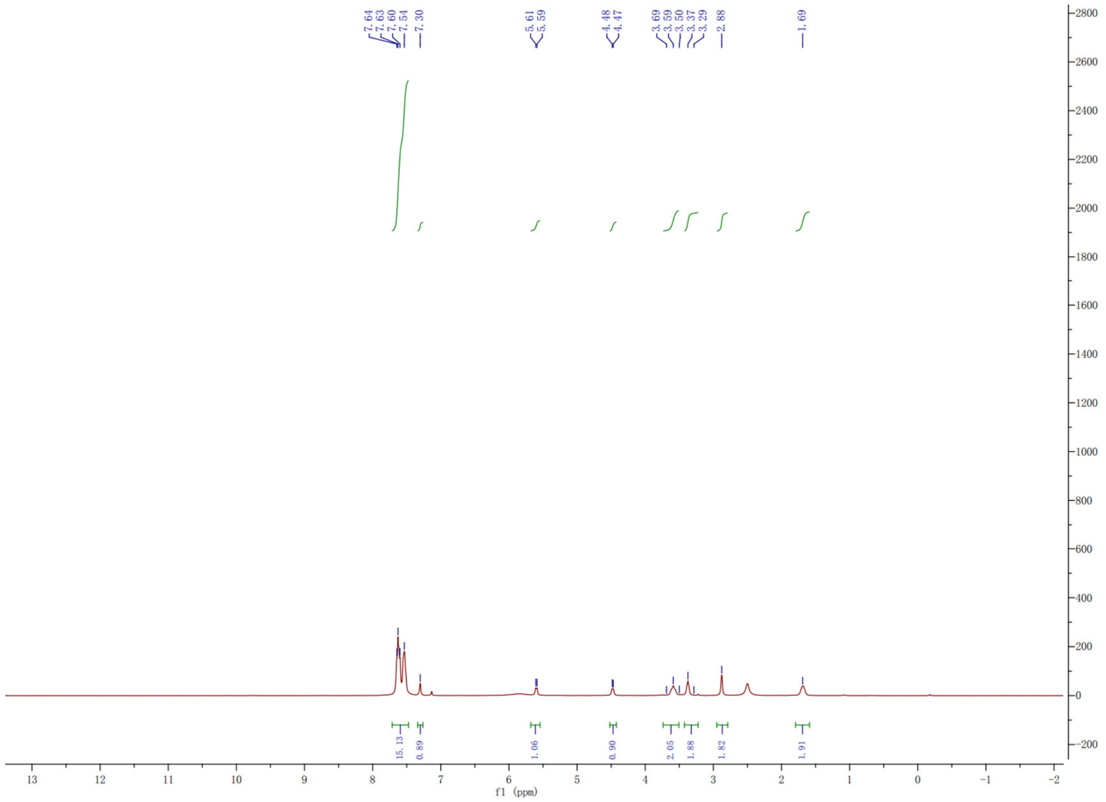

Figure S1. ^{1}H NMR (400MHz DMSO-d6). δ = 7.64-7.54 (m, 15H), 7.30 (s, 1H), 5.60 (d, J = 12Hz, 1H), 4.48- 4.47 (m, 1H), 3.69 - 3.50 (m, 2H), 3.37-3.29 (m, 2H), 2.68 (s, 2H), 1.69 (s, 2H).

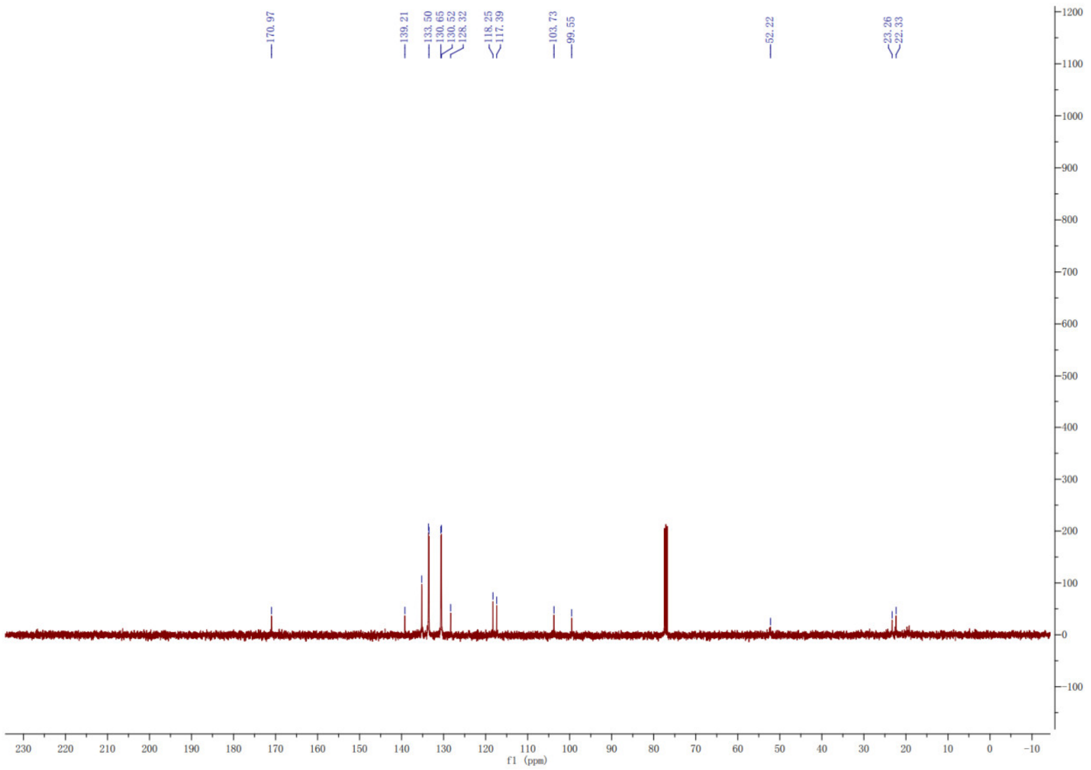

Figure S2. ^{13}C NMR (100MHz DMSO-d6). δ= 170.97, 139.21, 133.50, 130.65, 130.52, 128.32, 118.25, 117.39, 103.73, 99.55, 32.22, 23.26, 22.33. ESI-MS: 427.8, calcd for C27H28N2OP+, 427.51.

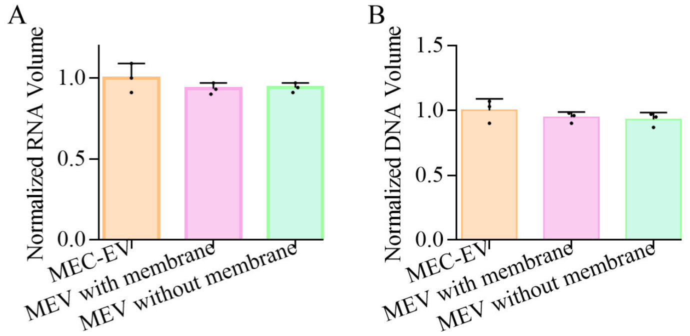

Figure S3. Total RNA and DNA content of MSC-EV and MEV. A,) Total RNA content of MSC-EV and MEV extruding with or without polycarbonate membranes was quantified and normalized. n=3. B), Total DNA content of MSC-EV and MEV extruding with or without polycarbonate membranes was quantified and normalized. n=3.

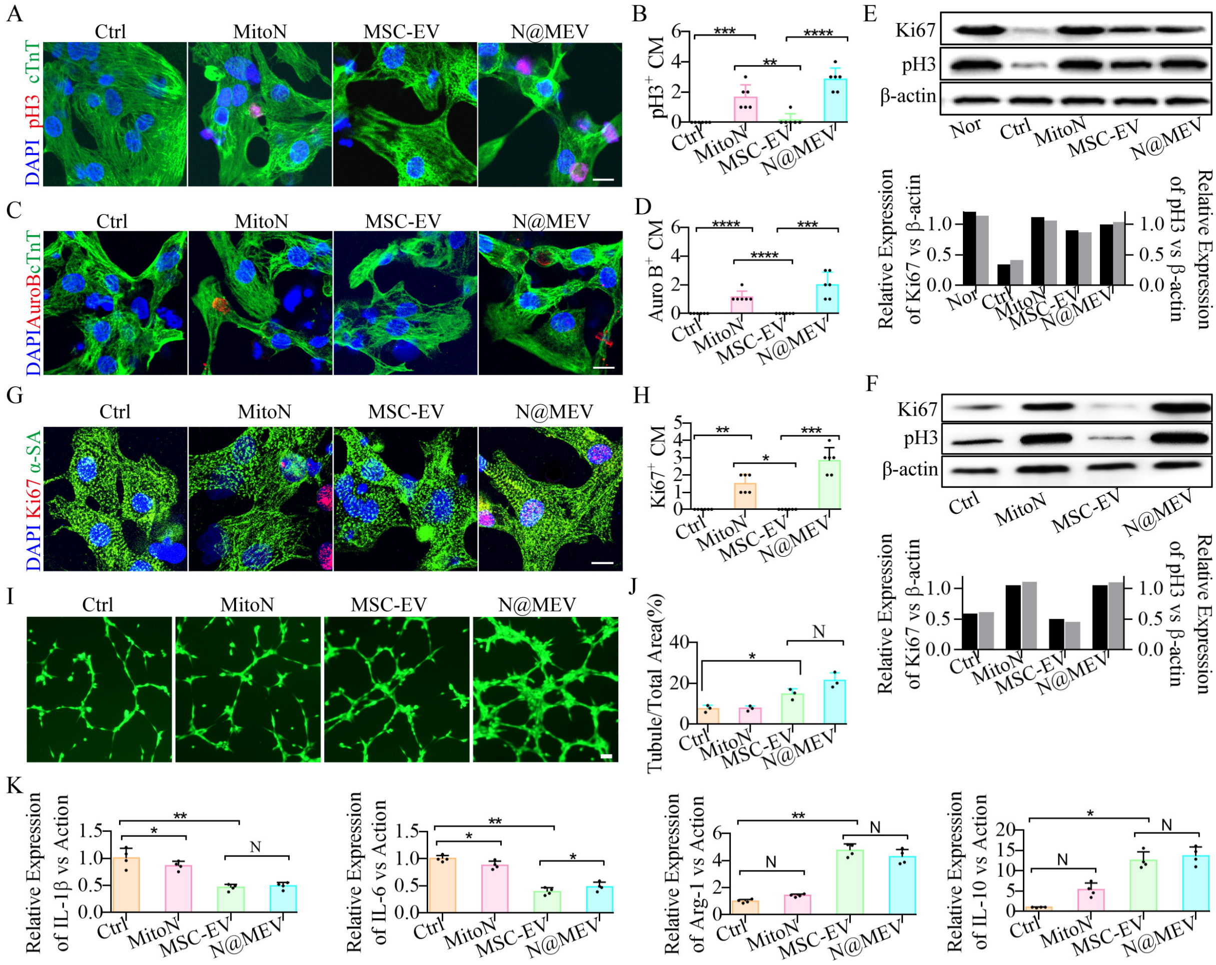

Figure S4. Neonatal cardiomyocyte proliferation after treatments. A, B), Immunofluorescent staining (A) and quantification (B) of P7 CM proliferation identified by pH3 after treatments. Green, cTnT; Red, pH3. bar=10μm, n=6. C, D), Immunofluorescent staining (C) and quantification (D) of P7 CM proliferation identified by Auro B. Green, cTnT; Red, Auro B. bar=10μm, n=6. E), Western blot (upper) and quantification (lower) determining Ki67 and pH3 expression of P1 CM after oxidative stress and treatments. F), Western blot (upper) and quantification (lower) determining Ki67 and pH3 expression of P7 CM after treatments. G, H), Immunofluorescent staining (G) and quantification (H) of P7 CM proliferation identified by Ki67 after H_2O_2-induced oxidative stress and treatments. Green, αSA; Red, Ki67. bar=10μm, n=6. I,J), Tubule formation images (I) and quantification (J) of HUVEC after treatments. Green, cells were stained with Calcein. bar=20μm, n=3. K), qRT-PCR analysis of M1 and M2-associated genes expression in macrophages after TNF-α stimulation and treatments. n=4. *$p<0.05$, **$p<0.01$, ***$p<0.001$, ****$p<0.0001$.

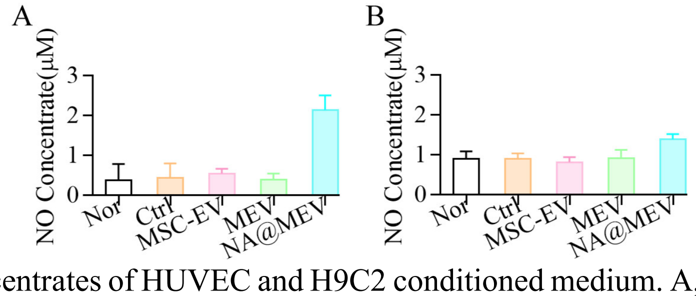

Figure S5. NO concentrates of HUVEC and H9C2 conditioned medium. A, B), NO concentrate was determined flowing respective formulations cocultured with HUVEC (A) and H9C2 (B) conditioned medium.

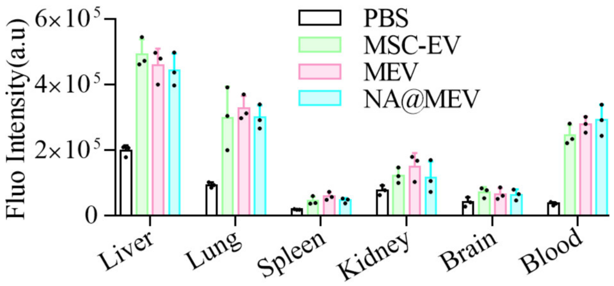

Figure S6. The distribution of respective formulations in other major organs. n=3.

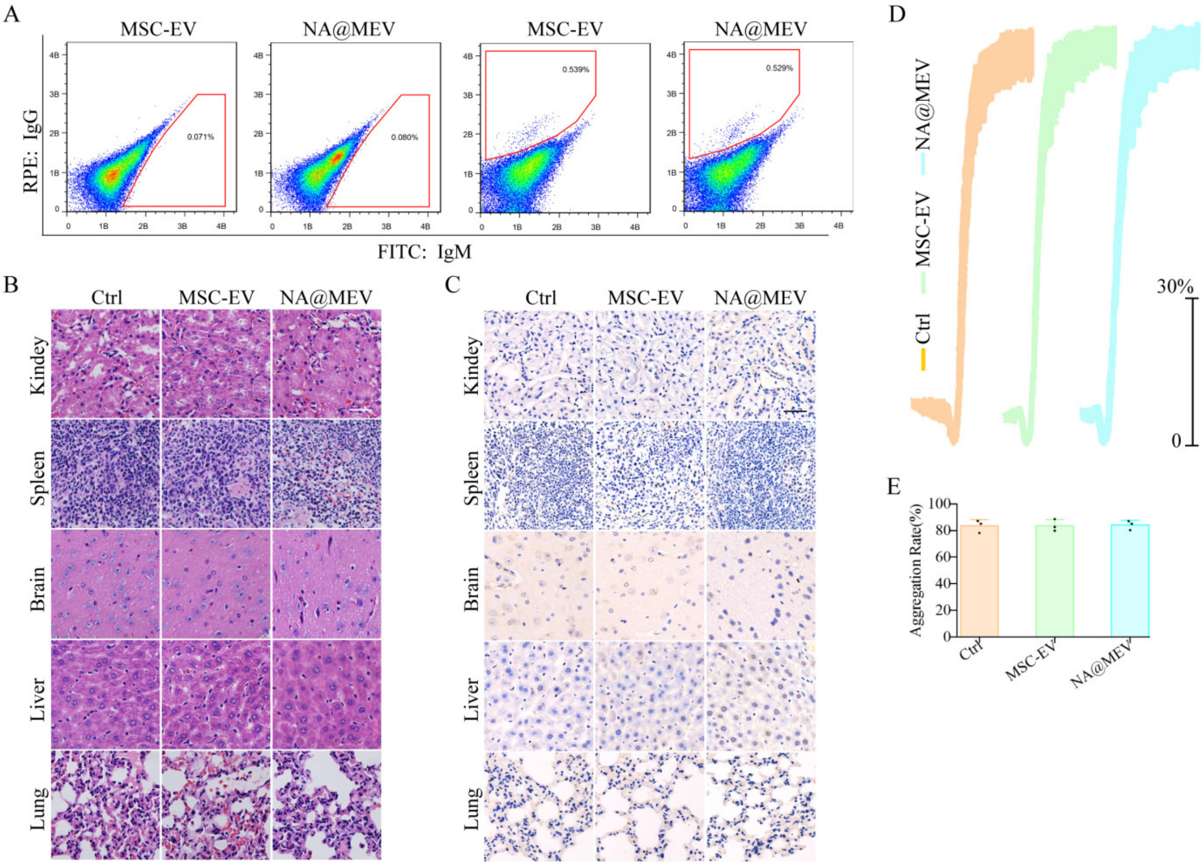

Figure S7. Biosafety of NA@MEV. A), Immune response to MSC-EV and NA@MEV 4 weeks after treatment was determined by flow cytometry. RPE: IgG; FITC: IgM. B,C), HE staining (B) and Caspase-3 IHC staining (C) of major organs showing none obvious histology changes after treatments. bar=50μm. D,E), Platelets aggregation after incubated with MSC-EV or NA@MEV was determined (D) and quantified (E) by lumiaggregometer. n=3.
